# Transjugular implantation of a leadless pacemaker in a 97-year-old patient with severe kyphosis and tortuous femoral veins: a case report

**DOI:** 10.3389/fcvm.2026.1932808

**Published:** 2026-10-15

**Authors:** Ming-Huan Fu, Xue-Fei Tao, Jie Gao, Yong Shi, Biao Cheng, Wei Zhang, Xiao-Han Chen

**Affiliations:** Department of Geriatric Cardiovascular Disease, Sichuan Provincial People’s Hospital, School of Medicine, University of Electronic Science and Technology of China, Chengdu, China

**Keywords:** cardiac pacing, leadless pacemaker, older adults, severe kyphosis, transjugular approach

## Abstract

**Background:**

Leadless pacemaker systems offer significant benefits for frail older adults by avoiding pocket- and lead-related complications. However, anatomical challenges including severe spinal deformities and significant venous tortuosity can complicate conventional transfemoral or trans-subclavian implantation.

**Case report:**

A 97-year-old man with severe kyphosis, tortuous femoral veins, and high-grade atrioventricular block was referred for permanent pacing. Supine positioning for subclavian access was not feasible, and the femoral anatomy posed an unacceptably high procedural risk. Following a multidisciplinary assessment and procedural planning, a transjugular approach was selected. Implantation was performed with the patient in the left lateral decubitus position, using adapted fluoroscopic projections and temporary femoral pacing as backup. An Aveir ventricular response leadless pacemaker was successfully delivered via the right internal jugular vein. Final device parameters were satisfactory, with a sensing amplitude of 8.5 mV, a pacing threshold of 0.5 V at 0.4 ms, and an impedance of 510 Ω. No perioperative complications occurred. At follow-up, exertional dyspnea had resolved, and device performance remained stable.

**Conclusion:**

This case demonstrates that transjugular implantation of a leadless pacemaker can be a safe and feasible alternative for older adults with complex anatomical limitations that contraindicate standard pacing approaches. Careful preprocedural assessment and adaptation of intraoperative techniques are essential for procedural success in high-risk settings.

## 1 Introduction

Leadless pacemaker technology has significantly improved the management of bradyarrhythmias, particularly in older adults and frail patients, by eliminating the need for subcutaneous generator pockets and transvenous leads. This design reduces the incidence of pocket-related infections, lead dislodgement, and vascular complications (1). Despite these advantages, the broader use of leadless systems may be limited by patient-specific anatomical factors, particularly tortuous or occluded femoral venous pathways, which are the standard route for device delivery (2). Moreover, pronounced spinal deformities, including kyphosis, can hinder the supine positioning necessary for trans-subclavian or axillary access, adding further complexity to pacemaker implantation.

**Table 1:** Leadless pacemaker interrogation parameters over time.

Parameter	Immediate postimplant	1-month follow-up	4-month follow-up
Sensing amplitude (mV)	8.5	8.2	8.7
Pacing threshold	0.5 V at 0.4 ms	0.5 V at 0.4 ms	0.5 V at 0.4 ms
Impedance (Ω)	510	530	530
Estimated longevity	-	-	14.4 years

**Table 2:** Functional and quality of life assessments.

Assessment tool	Preoperative	1-week follow-up	1-month follow-up	4-month follow-up
NYHA functional class	III	-	II	II
KCCQ overall score	18	22	27	26
6-min walk test	Unable	Unable	Unable	Unable

NYHA, New York Heart Association; KCCQ, Kansas City Cardiomyopathy Questionnaire.

This case describes a 97-year-old patient with severe kyphosis, tortuous femoral veins, and multiple comorbidities who required pacemaker therapy for symptomatic bradycardia. Due to contraindications to conventional implantation strategies, a transjugular approach for leadless pacemaker implantation was selected. This case emphasizes the need for individualized preprocedural assessment and procedural adaptation for patients with challenging anatomical and clinical profiles.

## 2 Case presentation

### 2.1 Demographics and clinical history

A 97-year-old man (born 10 September 1927) was admitted to Sichuan Provincial People's Hospital on 29 July 2025, for assessment of recurrent exertional dyspnea lasting approximately 2 years. Symptoms were triggered by routine physical activities, such as walking 10–20 m, and typically resolved after 5–10 min of rest. No episodes of syncope, dizziness, amaurosis, chest pain, palpitations, cough, expectoration, hemoptysis, orthopnea, or paroxysmal nocturnal dyspnea were reported.

The medical history included hypertension, atrial fibrillation (AF), chronic kidney disease (CKD), chronic obstructive pulmonary disease, osteoporosis, and cholecystolithiasis. No history of previous cardiac surgery or interventional procedures was documented.

### 2.2 Baseline characteristics

#### 2.2.1 Anthropometrics

The patient was 150 cm tall and weighed 40 kg, corresponding to a body mass index (BMI) of 17.7 kg/m^2^, classified as severe underweight (BMI < 18.5 kg/m^2^) (3).

#### 2.2.2 Cardiac function

Functional status was assessed as New York Heart Association (NYHA) class III (4).

#### 2.2.3 Frailty status

Assessment using the Frail Scale resulted in a score of 3, indicating moderate-to-severe frailty (5).

#### 2.2.4 Mobility

Wheelchair assistance was required for ambulation, and pronounced kyphosis hindered the ability to maintain a supine position (Figure 1).

**Figure 1:**
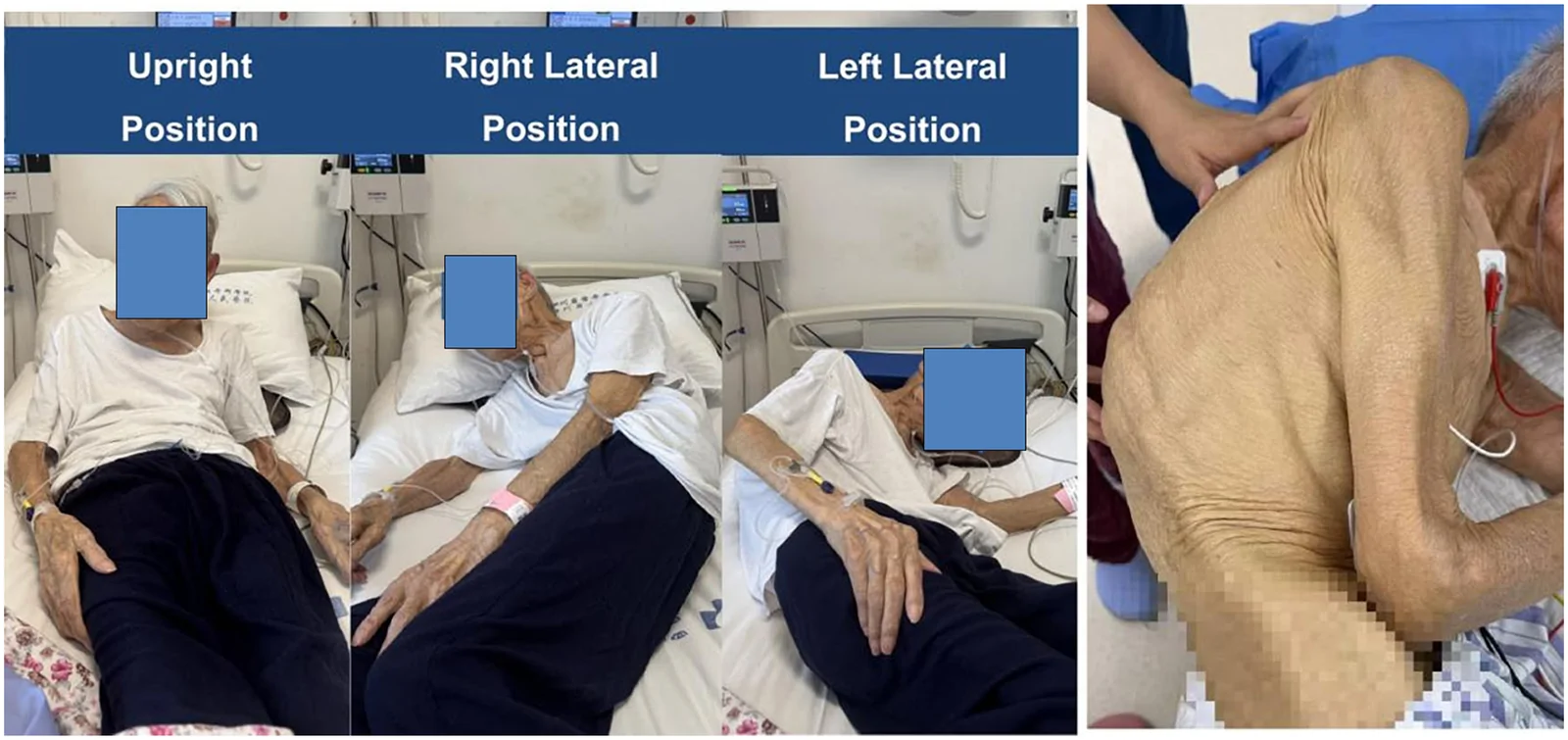
Severe thoracic kyphosis.

### 2.3 Diagnostic investigations

#### 2.3.1 Electrocardiography

After admission, an electrocardiogram examination on 15 August 2025, revealed atrial flutter with a ventricular rate of 37 beats per minute (Figure 2A).

**Figure 2:**
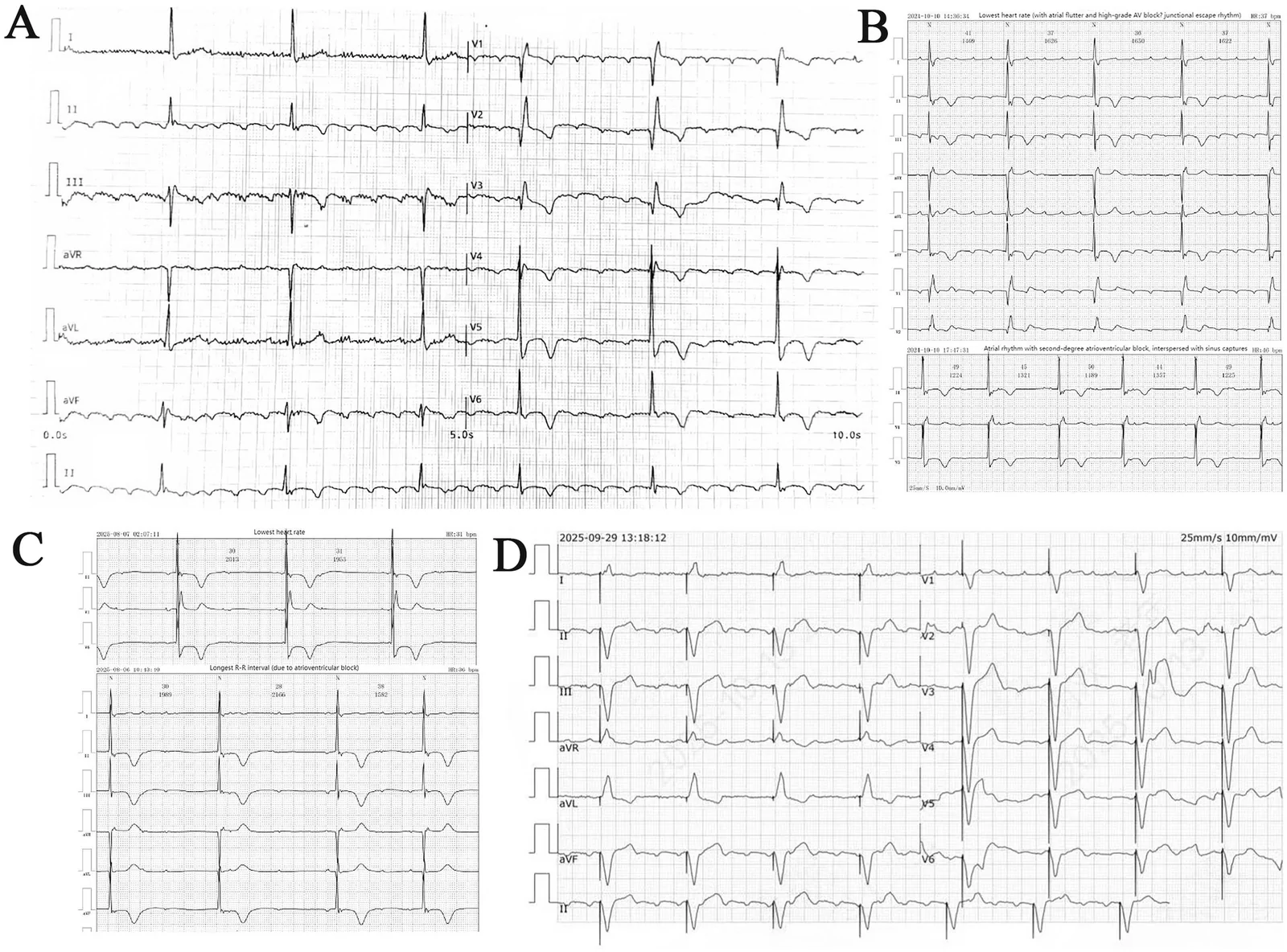
**(A)** Electrocardiogram recorded on 15 August 2025, **(B)** 24-h Holter monitoring conducted on 11 October 2024, **(C)** 24-h Holter monitoring conducted on 6 August 2025, and **(D)** electrocardiogram recorded on 29 September 2025.

#### 2.3.2 Twenty-four-hour Holter monitoring

Ambulatory monitoring on 11 October 2024, confirmed intermittent high-degree atrioventricular block, with the longest R–R interval exceeding 2.01 s (Figure 2B). Repeat monitoring on 6 August 2025, indicated persistent bradyarrhythmia without significant improvement (Figure 2C).

#### 2.3.3 Imaging studies

Thin-slice chest computed tomography (CT) on 14 July 2025, showed no evidence of pulmonary embolism or significant pulmonary parenchymal abnormalities. A chest radiograph on 18 August 2025, indicated pronounced thoracic kyphosis (Figure 3A). Duplex ultrasonography of the lower extremity veins on 19 August 2025, demonstrated marked bilateral femoral vein tortuosity with luminal irregularities consistent with chronic venous disease. Transthoracic echocardiography on 4 August 2025, showed preserved left ventricular systolic function, with an ejection fraction of 58%, mild mitral regurgitation, and no significant right ventricular dysfunction.

**Figure 3:**
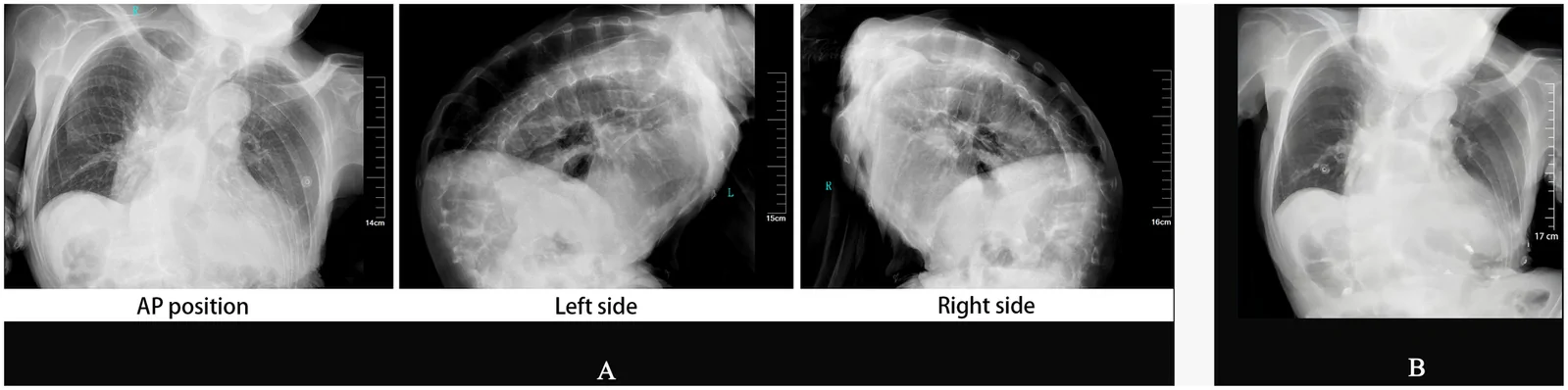
**(A)** Chest radiograph taken on 18 August 2025 and **(B)** chest radiograph taken on 18 September 2025.

#### 2.3.4 Laboratory findings

Laboratory testing on 19 August 2025, indicated a serum creatinine level of 152.4 μmol/L, corresponding to an estimated glomerular filtration rate of 29.3 mL/min/1.73 m^2^, consistent with stage 4 CKD (6). Serum electrolytes, complete blood count, and coagulation parameters were within normal reference ranges.

## 3 Management

### 3.1 Preprocedural planning

A multidisciplinary conference involving cardiac electrophysiologists, radiologists, anesthesiologists, and geriatric specialists was convened to evaluate feasible pacing strategies. Several options were reviewed.

Implantation of a trans-subclavian lead-based pacemaker was deemed unsuitable as the patient could not tolerate the supine position required for subclavian venous access. In addition, the patient’s profound underweight status and advanced frailty were considered to significantly increase the risk of pocket-related complications, including infection and hematoma formation (7–9).

A transfemoral approach for leadless pacemaker implantation was considered high-risk due to expected severe tortuosity of the femoral veins, which could significantly increase the likelihood of vascular injury, venous dissection, or suboptimal device positioning (10).

By contrast, a transjugular leadless pacemaker strategy was chosen as the most suitable alternative. Preprocedural ultrasonography confirmed patency of the right internal jugular vein (RIJV), providing a relatively short and straight trajectory to the right ventricle (RV) when the procedure was performed in the left lateral decubitus position (2).

Written informed consent was obtained from the patient and the legally authorized representative. The study protocol received approval from the Institutional Review Board of Sichuan Provincial People’s Hospital (approval number: SPH-IRB-2025-078).

### 3.2 Procedural details

The intervention was carried out on 26 August 2025, in the cardiac catheterization laboratory under local anesthesia using 2% lidocaine.

#### 3.2.1 Patient positioning

To accommodate the pronounced kyphotic deformity and facilitate access to the RIJV, the patient was positioned in the left lateral decubitus posture.

#### 3.2.2 Fluoroscopic guidance

Fluoroscopic projections were adjusted based on preprocedural analysis of chest CT images using FluoCT software. The optimal tangential views were identified as left anterior oblique 20° (Figures 4A–C) for right-sided visualization and left anterior oblique 80° (Figure 4D) for left-sided visualization, enhancing delineation of cardiac anatomy and accurate device positioning in the presence of thoracic deformity. The cumulative radiation dose during the procedure was recorded as 731 mGy.

**Figure 4:**
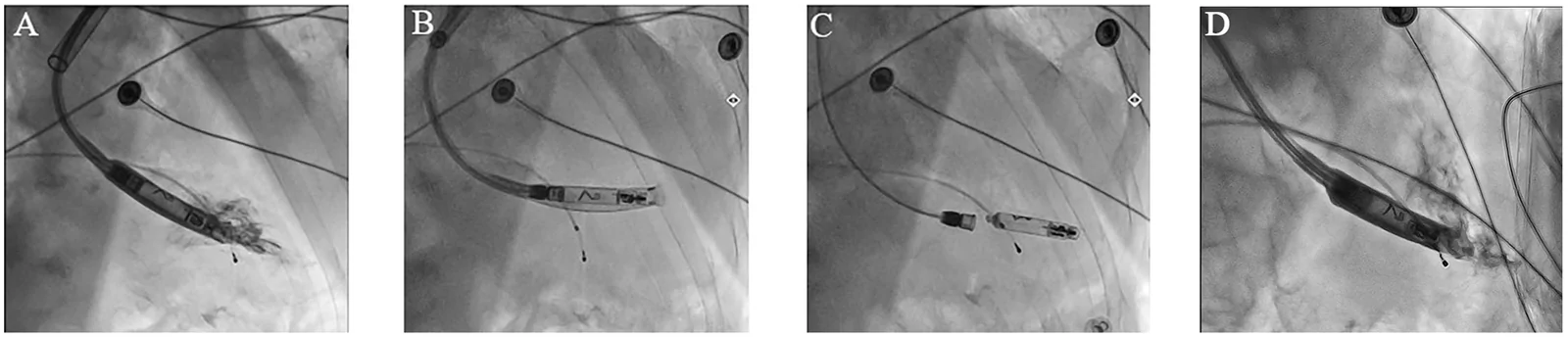
**(A–C)** Left anterior oblique 20° fluoroscopy images for right-sided visualization during operation and **(D)** left anterior oblique 80° fluoroscopy image for left-sided visualization during operation.

#### 3.2.3 Vascular access

To reduce the risk of profound bradycardia during the intervention, a temporary pacing lead was positioned via the right femoral vein as a backup; placement was completed in approximately 15 min. Ultrasound-guided cannulation of the RIJV was then performed, followed by insertion of a 6-French introducer sheath.

#### 3.2.4 Sheath dilation

The venous access tract was progressively dilated to accommodate a 27-French delivery sheath, which was smoothly advanced into the superior vena cava (SVC) without resistance.

#### 3.2.5 Device implantation

The delivery catheter for the Aveir ventricular response (VR) leadless pacemaker (Abbott Laboratories, Chicago, IL, USA) was introduced through the 27-French sheath, with the white handle oriented upwards. Telemetry was activated while the system was positioned in the SVC, after which the catheter was advanced across the tricuspid valve into the RV. Clockwise rotation facilitated passage through the tricuspid valve, whereas counterclockwise rotation directed the catheter toward the low septal region of the right ventricular wall.

#### 3.2.6 Position verification

RV angiography was performed in left anterior oblique projections of 20° and 80° and confirmed appropriate device placement, with adequate distance from the right ventricular apex and the anterior and posterior interventricular grooves. A total of 60 mL of iopamidol contrast medium was administered. Intracardiac mapping demonstrated a steep QRS slope, an S wave above the baseline, and 3 mV of ST-segment elevation, findings consistent with optimal myocardial contact (11).

#### 3.2.7 Fixation and release

Fixation was achieved by slow clockwise rotation of the delivery system at approximately one click per second, completing 1.25 cycles. Electrical testing at 0.5 and 1.0 rotations indicated persistent QRS widening and stable ST-segment elevation. A wobble test confirmed secure fixation. After confirming coaxial alignment between the docking cap and the device, the pacemaker was released by counterclockwise rotation of the release mechanism.

### 3.3 Procedural outcomes

#### 3.3.1 Device parameters

Final pacing parameters were satisfactory, with a sensing amplitude of 8.5 mV, a pacing threshold of 0.5 V at 0.4 ms, and an impedance of 510 Ω.

#### 3.3.2 Complications

No perioperative adverse events were observed, including vascular injury, bleeding, cardiac tamponade, or device malposition. Hemodynamic stability was maintained throughout the intervention, which lasted a total of 72 min.

#### 3.3.3 Postprocedural care

Following the procedure, the RIJV sheath was removed, and hemostasis was achieved with manual compression for 10 min. The patient was subsequently monitored in the cardiac intensive care unit for 24 h, during which no bleeding complications or clinically significant arrhythmias were detected. Intravenous hydration with 1,000 mL of normal saline was administered at a rate of 60 mL/h to prevent contrast-induced nephropathy. Rivaroxaban 10 mg was given for anticoagulation therapy. Hospital discharge occurred on the third day after the procedure.

### 3.4 Follow-up

Follow-up evaluations were conducted at 1 week, 1 month, and 4 months after device implantation.

#### 3.4.1 Clinical status

Exertional dyspnea had resolved during follow-up, and no episodes of syncope, dizziness, or symptoms indicative of heart failure were reported. Functional capacity improved, with NYHA classification advancing to class II.

#### 3.4.2 Device performance and projected longevity

At the 4-month assessment, the estimated device longevity was 14.4 years. Device interrogation demonstrated stable parameters, including a sensing amplitude of 8.7 mV, pulse amplitude of 2.0 V, and pulse width of 0.4 ms.

#### 3.4.3 Electrocardiographic and device assessment

At the 1-month postoperative visit, a surface electrocardiogram was obtained, as shown in Figure 2D. Device interrogation at that time indicated a sensing amplitude of 8.2 mV, a pacing threshold of 0.5 V at 0.4 ms, and an impedance of 530 Ω (Table 1), all within normal ranges.

#### 3.4.4 Imaging

Chest radiography performed at 1 month demonstrated stable device position without evidence of migration or thoracic complications (Figure 3B).

#### 3.4.5 Renal function

Follow-up renal function testing revealed no significant change in estimated glomerular filtration rate from baseline.

#### 3.4.6 Functional assessment and quality of life

The patient remained unable to ambulate independently and therefore could not complete the 6-minute walk test. Kansas City Cardiomyopathy Questionnaire scores increased from 18 points preoperatively to 22 points at 1 week, 27 points at 1 month, and 26 points at 4 months following implantation (Table 2), indicating an overall improvement in health-related quality of life.

## 4 Discussion

This case highlights the complex interplay of advanced age, severe anatomical distortion, and the need for safe and effective cardiac pacing, presenting a triad of challenges rarely encountered in routine practice. Successful implantation of a leadless pacemaker via the RIJV in a patient with profound kyphoscoliosis, who could tolerate only a strict left lateral decubitus position, illustrates the importance of technical flexibility and individualized procedural planning. Beyond demonstrating procedural feasibility, this case encourages broader consideration of mechanism-based strategy selection, comparative assessment of alternative access routes, and long-term management in patients with complex anatomical constraints.

Importantly, this report differs from previously described challenging leadless pacemaker implantations in that the spinal deformity was so severe that supine positioning was impossible. As a result, the entire procedure was performed in the lateral decubitus position. This exceptional constraint, not previously reported in the literature, directly influenced all subsequent procedural decisions and emphasizes the need for adaptable approaches in highly selected clinical scenarios.

### 4.1 Mechanism-driven access planning: beyond superficial landmarks

The selection of the RIJV as the access route was not a default choice but the result of a deliberate, data-driven evaluation incorporating CT-based anatomical analyses. Although severe femoral venous tortuosity identified on ultrasonography posed a significant limitation, the access strategy was determined through anticipatory assessment rather than intraoperative necessity. Preprocedural analysis of chest CT images with three-dimensional reconstruction software, such as FluoCT, facilitated simulation of catheter paths from multiple potential access sites.

This analysis indicated that a transfemoral route would require navigating multiple sharp angles within a markedly deformed thoracic cavity, increasing the risk of endothelial injury, delivery system instability, and procedural failure. In contrast, the path from the RIJV, while less conventional for pacing procedures, offered a shorter and more direct route to the SVC and right atrium, albeit with a different angulation profile. The development of this individualized, anatomy-based vascular “roadmap” represents a shift from merely selecting an alternative access site when standard routes are unsuitable to adopting a precision-based approach to vascular access. In this paradigm, procedural planning is informed by predicted ease of navigation and safety based on patient-specific anatomical characteristics.

### 4.2 Comprehensive alternative analysis and justification for leadless single-chamber pacing

A thorough assessment of all potential therapeutic strategies was essential to management. Several alternatives were considered. First, a transfemoral leadless pacing approach requiring extensive catheter manipulation was deemed high-risk due to the confirmed severity of femoral venous tortuosity (2). Second, epicardial surgical pacing was considered unsuitable, given the high perioperative risks associated with advanced age, frailty, and multiple comorbidities. Third, pharmacological therapy alone was inadequate to address symptomatic and profound bradycardia. Finally, implantation of a conventional transvenous pacemaker via the subclavian approach was contraindicated, as supine positioning for pectoral pocket creation was not feasible. In addition, the markedly distorted and mobile thoracic cage was expected to increase the long-term risk of rib fracture and tricuspid valve interference (12).

The selection of the single-chamber RV leadless pacemaker (Aveir VR) resulted from a structured risk–benefit assessment that emphasized procedural minimalism, anatomical compatibility, and clinical indication. A documented history of AF and flutter eliminated the need for atrial sensing or tracking, meeting guideline-based criteria for VVI pacing (13). In addition, the leadless system avoided the long-term complications associated with conventional leads, such as lead dislodgment, tricuspid regurgitation, and venous occlusion. These risks are particularly relevant in patients with severe skeletal deformities, where implanted leads may be subjected to atypical mechanical forces. This management strategy is consistent with growing evidence supporting leadless pacemakers as a superior option for patients with limited vascular access or an increased risk of device-related infection (1).

### 4.3 Anticipating and mitigating long-term complication risks of the transjugular route

While the perioperative feasibility and safety of transjugular access have been established, careful consideration of potential long-term implications remains crucial for informed management. Anticipated chronic risks include device migration or embolization toward the pulmonary artery, which is uncommon given secure septal fixation and the anchoring mechanism of the device tines; thrombus formation on the device with a subsequent risk of pulmonary embolism; and delayed vascular complications at the jugular access site, such as venous stenosis or thrombosis. The latter is considered less likely than with femoral access because of the higher flow in the internal jugular vein. In addition, pericardial effusion or cardiac perforation remains an inherent, though infrequent, risk associated with all leadless pacemaker implantations (9, 12).

In this patient, the left lateral decubitus position and cranio-caudal trajectory achieved through the RIJV may have positively influenced implantation mechanics, potentially promoting stable engagement with the right ventricular septum. A structured long-term surveillance strategy has been implemented, including periodic transthoracic echocardiography to monitor RV function and device position, as well as cervical venous Doppler ultrasonography to assess venous patency. Such proactive monitoring is a central component of ongoing management when using this less conventional access route.

### 4.4 Positioning, imaging, and technical uniqueness: a paradigm shift

A defining feature of this intervention was the mandatory use of the left lateral decubitus position, necessitating a fundamental recalibration of standard fluoroscopic paradigms. Conventional right and left anterior oblique projections are based on supine anatomy and were not directly applicable in this context. To address this limitation, preprocedural CT-based three-dimensional reconstruction was used to derive patient-specific fluoroscopic left anterior oblique projections of 20° and 80°, approximating functional equivalents of right and left anterior oblique views, respectively, compensating for axial body rotation and enabling accurate visualization of the tricuspid valve plane and right ventricular septum.

This approach represents a significant advancement beyond empirically adjusting imaging angles during the procedure, reflecting a deliberate, image-guided strategy tailored to individual anatomy. The temporary femoral pacing system served a dual purpose: it provided hemodynamic support during the intervention and acted as a practical assessment tool, further confirming the impracticality of femoral venous access for permanent device delivery.

### 4.5 Contrasting with published literature and highlighting distinctive value

Comparisons with previously reported complex leadless pacemaker implantations highlight the unique nature of this case. Earlier studies have described transfemoral implantation in patients with mild-to-moderate spinal deformities or transjugular approaches performed with patients in the supine position. In contrast, this study incorporates three uncommon features: severe skeletal deformity that precluded any degree of supine positioning, use of the RIJV as the access route, and completion of the entire procedure in the left lateral decubitus position. This specific combination has not been previously documented.

By demonstrating procedural success under these constraints, this case expands the understanding of feasibility in leadless pacing. It shows that neither advanced kyphoscoliosis nor an inability to tolerate a supine posture should be considered absolute contraindications. Furthermore, it presents a reproducible procedural framework, including preprocedural CT-based planning, individualized access selection, and adapted fluoroscopic techniques, which may inform the management of similarly complex cases in patients with limited therapeutic options.

### 4.6 Broader clinical implications and future directions

This experience supports the routine use of advanced preprocedural imaging, such as CT or magnetic resonance imaging with three-dimensional reconstruction, in the planning workflow for anticipated complex device implantations. It also reinforces the RIJV as a viable and, in selected cases, potentially preferable access route. Future studies should prioritize multicenter registries to assess long-term outcomes of transjugular leadless pacing, focusing on sustained electrical performance, late complication rates, and comparative effectiveness relative to the transfemoral approach in populations with challenging anatomy. Furthermore, continued refinement of delivery systems, including the development of more flexible sheaths, may facilitate these non-standard implantation strategies.

## 5 Conclusion

This case presents an alternative strategy for implanting leadless pacemakers in patients with severe kyphosis and complex vascular anatomy. Transjugular delivery of a leadless pacemaker is a safe and effective alternative for older adults and frail patients with severe anatomical constraints, such as kyphosis and tortuous femoral veins, that preclude conventional pacing approaches. Procedural success depends on comprehensive preprocedural planning, including multidisciplinary evaluation, detailed vascular imaging, and tailored technical modifications like individualized positioning and fluoroscopic angulation, along with meticulous intraprocedural execution. Overall, this report emphasizes the importance of personalized, anatomy-driven decision-making in complex cardiac electrophysiology and supports broader consideration of transjugular leadless pacing in carefully selected high-risk populations.

## Data Availability

The raw data supporting the conclusions of this article will be made available by the authors, without undue reservation.

## References

[1] eurskens NE Tjong FV Knops RE. End-of-life management of leadless cardiac pacemaker therapy. Arrhythm Electrophysiol Rev. (2017) 6(3):129–33. 10.15420/aer.2017:16:1 29018521 PMC5614751

[2] Park S Kim J Park H Kim YC Kim MJ Kim JY. Comparison of transjugular and transfemoral approaches for leadless pacemaker implantation in Asian patients: an exploratory study. J Interv Card Electrophysiol. (2025) 69:411–8. 10.1007/s10840-025-02159-9 41231343

[3] Cruz-Jentoft AJ Bahat G Bauer J Boirie Y Bruyère O Cederholm T. Sarcopenia: Revised European Consensus on Definition and Diagnosis. Age Ageing. (2019) 48(1):16–31. 10.1093/ageing/afy169 30312372 PMC6322506

[4] Heidenreich PA Bozkurt B Aguilar D Allen LA Byun JJ Colvin MM. 2022 AHA/ACC/HFSA guideline for the management of heart failure: a report of the American College of Cardiology/American Heart Association joint committee on clinical practice guidelines. Circulation. (2022) 145(18):e895–1032. 10.1161/CIR.0000000000001063 35363499

[5] Abellan van Kan G Rolland Y Bergman H Morley JE Kritchevsky SB Vellas B. The I.A.N.A task force on frailty assessment of older people in clinical practice. J Nutr Health Aging. (2008) 12(1):29–37. 10.1007/BF02982161 18165842 PMC12892609

[6] Chen TK Knicely DH Grams ME. Chronic kidney disease diagnosis and management: a review. JAMA. (2019) 322(13):1294–304. 10.1001/jama.2019.14745 31573641 PMC7015670

[7] McGuire C Naitore J Ramu V. Pacemaker pocket erosion: a critical issue requiring immediate attention. Cureus. (2024) 16(12):e75581. 10.7759/cureus.75581 39803029 PMC11724444

[8] Hsu JC Varosy PD Bao H Wang Y Curtis JP Marcus GM. Low body mass index but not obesity is associated with in-hospital adverse events and mortality among implantable cardioverter-defibrillator recipients: insights from the National Cardiovascular Data Registry. J Am Heart Assoc. (2012) 1(6):e003863. 10.1161/JAHA.112.003863 23316325 PMC3540679

[9] Pagan E Gabriels J Khodak A Chang D Beldner S Epstein LM. Safety of leadless pacemaker implantation in the very elderly. Heart Rhythm. (2020 Dec) 17(12):2023–8. 10.1016/j.hrthm.2020.05.022 32454218

[10] Oliveira SF Carvalho MM Adão L Nunes JP. Clinical outcomes of leadless pacemaker: a systematic review. Minerva Cardiol Angiol. (2021) 69(3):346–57. 10.23736/S2724-5683.20.05244-5 32657558

[11] Lee JZ Mulpuru SK Shen WK. Leadless pacemaker: performance and complications. Trends Cardiovasc Med. (2018) 28(2):130–41. 10.1016/j.tcm.2017.08.001 28826669

[12] Dai H Liu H Gao C Han J Meng J Liu P. Comparison of postoperative outcomes between leadless and conventional transvenous pacemakers implantation: an up-to-date meta-analysis. Rev Cardiovasc Med. (2024) 25(10):359. 10.31083/j.rcm2510359 39484120 PMC11522773

[13] Pollak A Falk RH. Pacemaker therapy in patients with atrial fibrillation. Am Heart J. (1993) 125(3):824–30. 10.1016/0002-8703(93)90177-b 8094937

